# Rare Presentation of Leiomyoma of the Pancreas

**DOI:** 10.31486/toj.24.0034

**Published:** 2024

**Authors:** Laveena Balani, Somanath Malage, Rahul, Ashish Singh, Nalini Kanta Ghosh, Neha Nigam

**Affiliations:** ^1^ Department of Surgical Gastroenterology, Sanjay Gandhi Postgraduate Institute of Medical Sciences, Lucknow, India; ^2^ Department of Pathology, Sanjay Gandhi Postgraduate Institute of Medical Sciences, Lucknow, India

**Keywords:** *Anemia*, *jaundice*, *leiomyoma*, *pancreas*, *pancreaticoduodenectomy*

## Abstract

**Background:** Leiomyomas are benign neoplasms that originate from smooth muscle cells and most commonly occur in soft tissues, the gastrointestinal tract, and the uterus. Leiomyomas of the pancreas are exceptionally rare, with, to our knowledge, only 6 documented cases prior to this report. This case was also challenging because of the young age of the patient and an atypical initial presentation.

**Case Report:** A 21-year-old male presented with progressive jaundice and severe anemia. Contrast-enhanced computed tomography revealed a heterogeneously hyperenhancing mass in the arterial phase in the head of the pancreas with a double-duct sign. Endoscopic biliary drainage was performed, followed by endoscopic biopsy that revealed a mesenchymal tumor; leiomyoma was confirmed with an immunohistochemical evaluation. The patient underwent pancreaticoduodenectomy, and the histopathologic and immunohistochemical examinations of the resected specimen confirmed the diagnosis of a low-grade leiomyoma arising from the ampulla of Vater.

**Conclusion:** This case not only highlights the rarity of pancreatic leiomyomas and the potential for atypical presentation but also emphasizes the importance of considering leiomyoma in the differential diagnosis, even in young patients, and supports surgical resection as the preferred treatment approach.

## INTRODUCTION

Leiomyoma, a benign neoplasm originating from smooth muscle cells, is well documented to occur in soft tissues, the gastrointestinal tract, and the uterus. However, leiomyoma arising from the pancreas is exceedingly rare with, to our knowledge, only 6 cases reported in the literature as of April 2024.^1-6^ We add our case to the limited existing literature on pancreatic leiomyomas and discuss clinical significance, diagnostic considerations, and potential therapeutic interventions.

## CASE REPORT

A 21-year-old male presented with symptoms of painless progressive jaundice and severe anemia for 1 month. He had no history of melena, and the family history was not remarkable. The patient was icteric, but no abnormality was obvious from the abdominal examination. Laboratory workup showed hemoglobin of 7.7 g/dL (reference range, 14-17.5 g/dL), total bilirubin of 10.1 mg/dL (reference range, 0.1-1.3 mg/dL), direct bilirubin of 6.7 mg/dL (reference range, 0-0.4 mg/dL), aspartate aminotransferase of 214 U/L (reference range, 5-40 U/L), alanine aminotransferase of 328 U/L (reference range, 5-40 U/L), alkaline phosphatase of 397 U/L (reference range, 35-150 U/L), and serum albumin of 4.2 g/dL (reference range, 3.5-5.5 g/dL). Serum levels of carcinoembryonic antigen, carbohydrate antigen 19.9, and chromogranin were 2.05 ng/mL (reference range, 0-5 ng/mL), 232 U/mL (reference range, 0-37 U/mL), and 26 ng/mL (reference value, <100 ng/mL), respectively.

**Figure 1.**
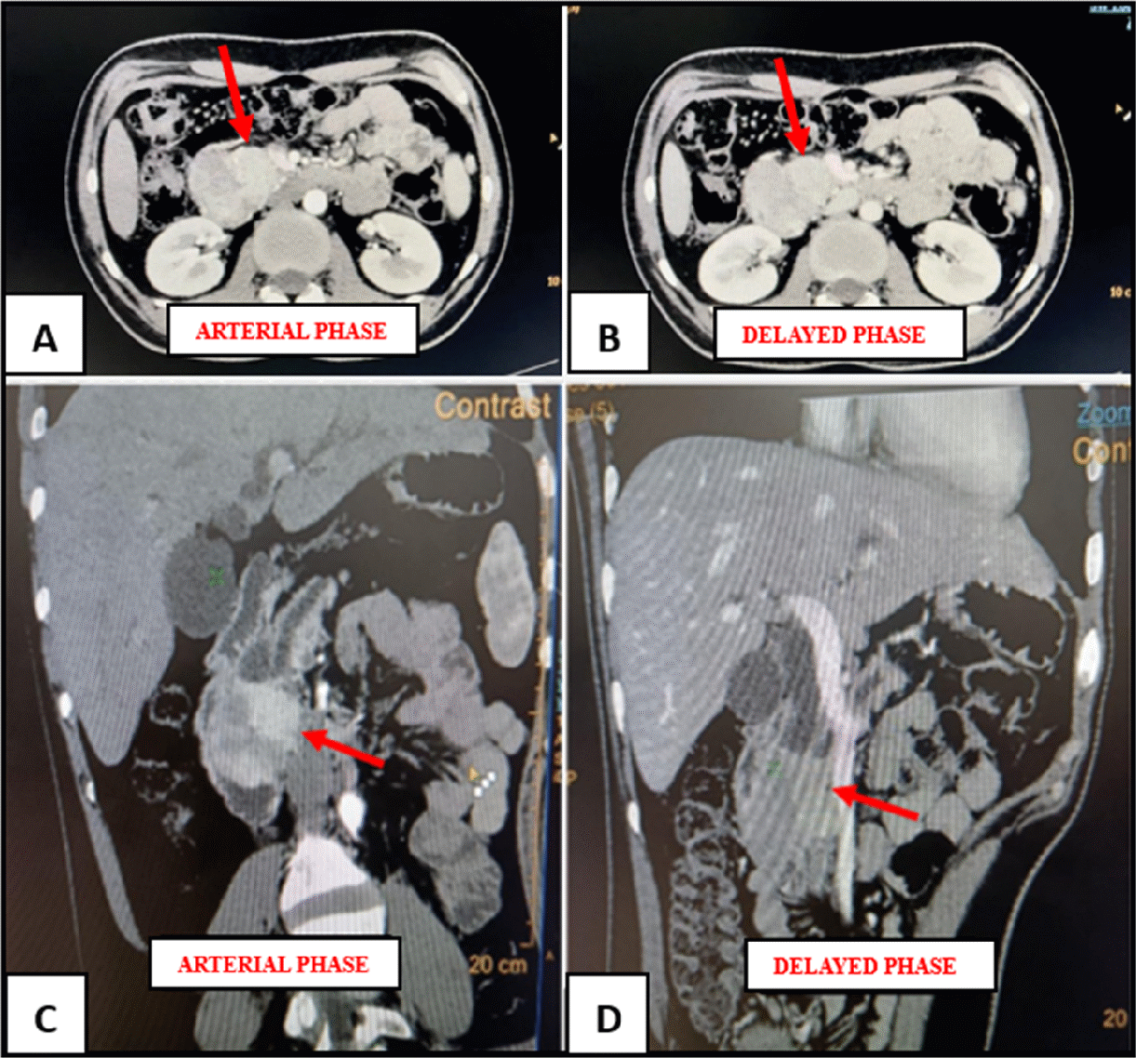
(A) Axial and (C) coronal views of the heterogeneously hyperenhancing periampullary mass in the arterial phase. (B) Axial and (D) coronal views of the isoenhancing periampullary mass in the delayed phase.

Contrast-enhanced computed tomography (CT) of the abdomen showed a 5.5-cm heterogeneously hyperenhancing mass in the head of the pancreas in the arterial phase that was isoenhancing in the delayed phase, with dilated main pancreatic duct and common bile duct (double-duct sign) (Figure 1). Upper gastrointestinal endoscopy showed an ulceroproliferative lesion in D1 and D2 with friable mucosa. Biopsy was suggestive of a mesenchymal lesion. On immunohistochemical evaluation, tumor cells were positive for vimentin, smooth muscle actin, and desmin and negative for CD117, DOG1, and CD34. The Ki-67 proliferative index was 8% to 10%.

Preoperative endoscopic biliary drainage was performed, and iron supplementation (2 injections of ferric carboxymaltose 750 mg, 7 days apart) was administered to correct anemia. After resolution of his anemia, the patient underwent open pancreaticoduodenectomy and had an uneventful postoperative recovery.

**Figure 2.**
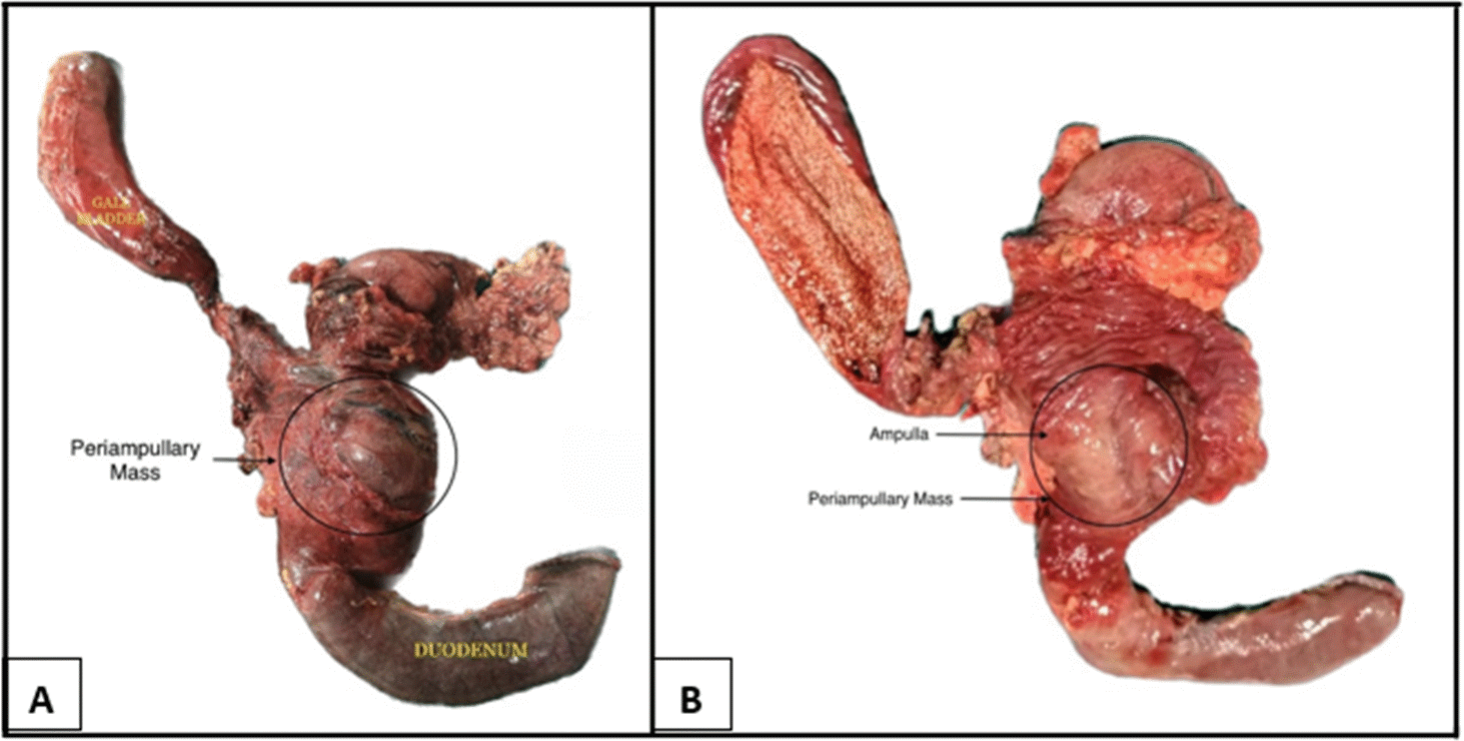
(A) Excised specimen from the pancreaticoduodenectomy. (B) Dissected specimen shows periampullary mass with normal duodenal mucosa.

**Figure 3.**
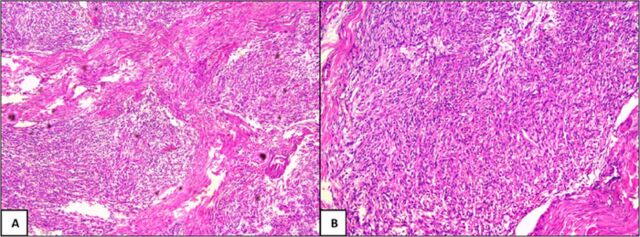
(A) Tumor cells were arranged in intersecting fascicles and bundles and (B) showed bland spindle cells with oval to elongated nuclei, dispersed chromatin, inconspicuous nucleoli, and a moderate amount of eosinophilic cytoplasm.

**Figure 4.**
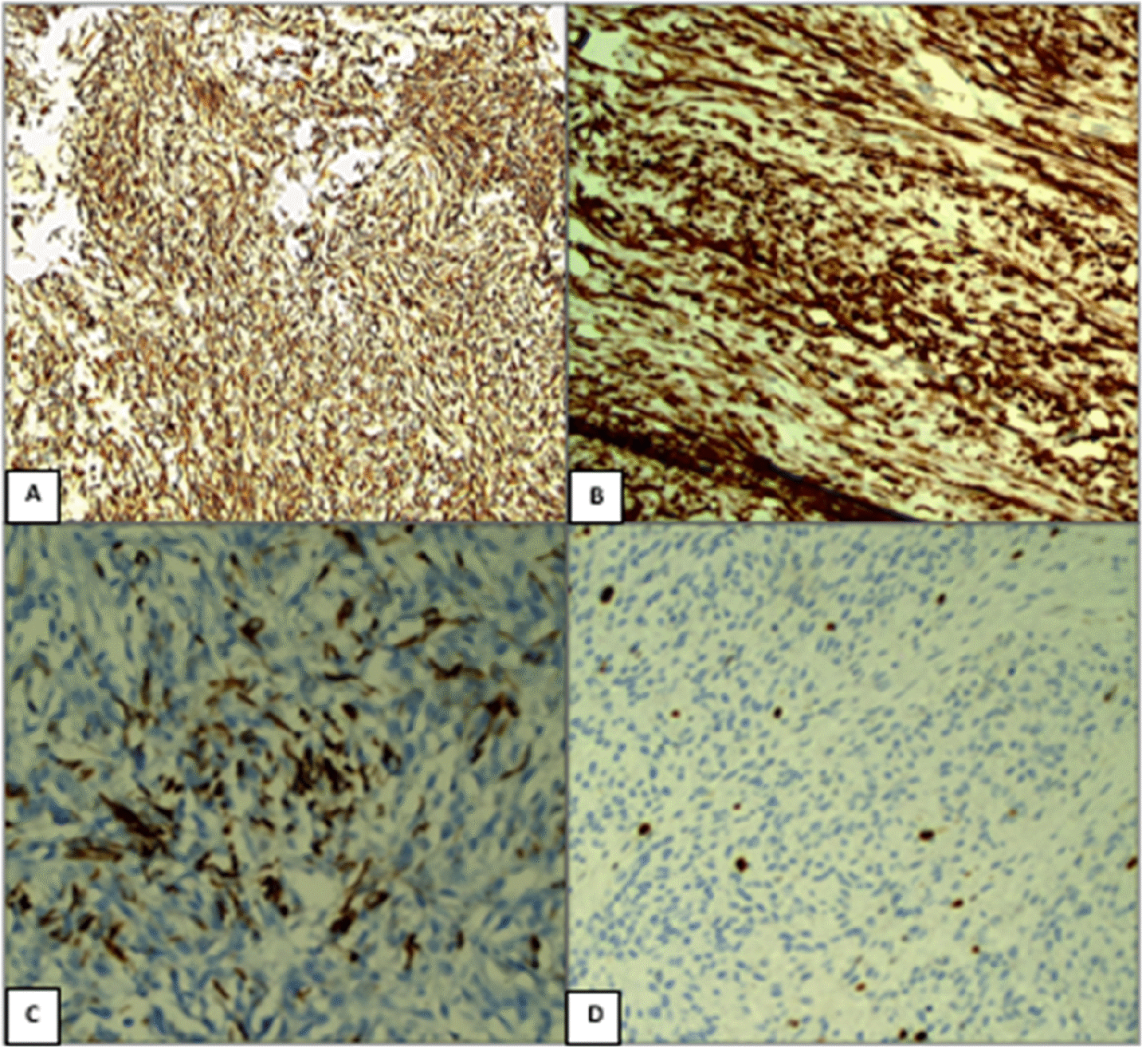
On immunohistochemical evaluation, the tumor cells were diffusely positive for (A) vimentin, (B) smooth muscle actin, and (C) desmin. (D) The Ki-67 proliferation index was ∼4% to 5%.

The resected specimen contained a 7-cm × 5-cm infiltrating tumor that involved the pancreas, bile duct, and duodenum (Figure 2). Histopathologic examination of the tumor cells showed bland spindle cells with oval to elongated nuclei, dispersed chromatin, inconspicuous nucleoli, and a moderate amount of eosinophilic cytoplasm. Tumor cells were arranged in intersecting fascicles and bundles (Figure 3). On immunohistochemical evaluation, the tumor cells were diffusely positive for vimentin, smooth muscle actin, and desmin and negative for S100, CD117, and DOG1 (Figure 4). The Ki-67 proliferation index was ∼4% to 5%. All of these features are consistent with low-grade leiomyoma arising from the ampulla of Vater.

At 1-year follow-up, the patient was doing well and had no evidence of recurrence.

## DISCUSSION

The pancreas does not contain smooth muscle tissue, and pancreatic leiomyomas are thought to arise from the pancreatic duct and peripancreatic blood vessels.^7^ Because of the rare occurrence of pancreatic leiomyomas, diagnosis and appropriate management are challenging. To our knowledge, our case is only the seventh to be reported, and our patient is the youngest at 21 years (Table).^1-6^ The ages of the previously reported patients ranged from 31 to 75 years, demonstrating a broad age spectrum, and the sex distribution was equal, with 3 males and 3 females.

**Table.** Leiomyoma of the Pancreas Case Reports

Study	Age, years/Sex	Clinical Presentation	Site in Pancreas/Size, cm	Radiologic Findings	Treatment	Outcome/Duration of Follow-up
Nakamura et al, 2000^1^	72/F	Incidentally diagnosed	Head/5.5	CECT: Hyperenhancing in arterial phase, isoenhancing in delayed phase	Enucleation	NR
Wisniewski et al, 2006^2^	52/M	Incidentally diagnosed	Head/2.5	CECT: Hypoenhancing in arterial phase, hyperenhancing in delayed phase	Pancreaticoduodenectomy	No recurrence/1 year
				EUS: Hypoechoic		
Sato et al, 2012^4^	62/F	Incidentally diagnosed	Head/3.5	CECT, EUS: Hypervascular tumor	Observation	NR
Kant et al, 2021^3^	75/M	Dyspeptic symptoms	Head/4	CECT: Peripheral enhancement in arterial phase and isoenhancing in delayed phase	Observation	Conversion to malignancy/13 years
Luo et al, 2022^5^	74/M	Incidentally diagnosed	Uncinate process/4	CECT: Hypoenhancing	Laparoscopic partial pancreatectomy	No recurrence/6 months
				MRI: T1 hypointense, T2 hyperintense		
Petchpiboolthai et al, 2023^6^	31/F	Abdominal pain	Head/NR	CECT: Heterogeneously enhancing	Pancreaticoduodenectomy	NR
Present case	21/M	Obstructive jaundice, anemia	Head/7	CECT: Hyperenhancing in arterial phase, isoenhancing in delayed phase	Pancreaticoduodenectomy	No recurrence/1 year

CECT, contrast-enhanced computed tomography; EUS, endoscopic ultrasound; F, female; M, male; MRI, magnetic resonance imaging; NR, not reported.

The clinical presentation of leiomyoma is varied. In the reported cases, 4 lesions were detected incidentally, and 2 patients presented with dyspepsia and vague abdominal pain. Our patient presented with obstructive jaundice and anemia, leading us to consider the usual differential diagnosis such as surgical obstructive jaundice. All the previously reported patients had lesions located in the head or uncinate process of the pancreas, as did our patient, with lesion sizes ranging from 2.5 cm to 5.5 cm (7 cm in our case).

On radiologic evaluation with contrast-enhanced CT, the lesion is heterogeneously hyperenhancing in the arterial phase and isoenhancing in the delayed phase. On magnetic resonance imaging, the lesion appears hypointense on T1-weighted images, with high arterial phase enhancement following gadolinium injection, and hyperintense on T2-weighted image.^5^ This enhancement pattern resembles that of neuroendocrine tumors, so neuroendocrine tumors can commonly be considered in the preoperative diagnosis as they occur more frequently than pancreatic leiomyomas.

The management of a preoperatively diagnosed, incidentally detected pancreatic leiomyoma is not established. Sato et al suggested that observation alone is sufficient but did not provide long-term follow-up results for their patient,^4^ and Kant et al reported malignant transformation after 13 years of observation.^3^ Three cases reported surgical excision: 2 pancreaticoduodenectomies^2,6^ and 1 partial pancreatectomy.^5^ Nakamura et al reported that their patient underwent enucleation of the lesion because of the small size of the lesion and its distance from the pancreatic duct,^1^ but this procedure is typically recommended for endocrine tumors, serous or mucinous cystadenomas, solid pseudopapillary tumors, branch duct-intraductal papillary mucinous neoplasms of the pancreas, and other benign lesions.

Surgical resection remains the primary treatment for pancreatic leiomyoma for 2 primary reasons: (1) diagnostic uncertainty because of the unavailability of a pathology diagnosis in many cases, and (2) the risk of transformation to malignancy, as observed in Kant et al.^3^

Grossly, the leiomyoma is firm in consistency and has a smooth surface. Microscopically, as in our case, the tumor displays bland spindle cells arranged in interlacing fascicles and bundles. The spindle cells have oval to elongated nuclei, dispersed chromatin, inconspicuous nucleoli, and a moderate amount of eosinophilic cytoplasm. On immunohistochemical evaluation, leiomyomas show positive staining for vimentin, smooth muscle actin, and desmin. The presence of these markers aids in distinguishing leiomyomas from other mesenchymal lesions, such as gastrointestinal stromal tumors and schwannomas, and from neuroendocrine lesions. The Ki-67 proliferation index provides insight into the tumor's proliferative activity; the patient reported by Kant et al had a Ki-67 score of 20% after malignant transformation.^3^ In our case, despite a low Ki-67 score, the tumor was infiltrating the common bile duct and duodenum, suggesting malignancy.

## CONCLUSION

Pancreatic leiomyomas, being rare entities, present challenges in both diagnosis and management, and our case is noteworthy for the patient's initial presentation with obstructive jaundice, typically indicative of infiltration observed with malignancies. The diagnosis of pancreatic leiomyoma is primarily reliant on histopathologic and immunohistochemical analyses. Surgical resection is the preferred approach, given the uncertain nature of the tumors and evidence to suggest malignant conversion.

## Notes

*This article meets the Accreditation Council for Graduate Medical Education and the American Board of Medical Specialties Maintenance of Certification competencies for Patient Care and Medical Knowledge.*
